# Editorial for the Special Issue “New Knowledge in the Study of Coronaviruses: Towards One Health and Whole Genome Sequencing Approaches, 2nd Edition”

**DOI:** 10.3390/microorganisms14010216

**Published:** 2026-01-17

**Authors:** Simone Peletto

**Affiliations:** Istituto Zooprofilattico Sperimentale del Piemonte, Liguria e Valle d’Aosta, Via Bologna 148, 10154 Torino, Italy; simone.peletto@izsplv.it

The study of coronaviruses has undergone unprecedented acceleration over recent years, driven largely by the global impact of SARS-CoV-2 and growing recognition of the extraordinary diversity and zoonotic potential of coronaviruses across species. Advances in molecular biology, high-throughput sequencing, and bioinformatics have substantially improved our ability to detect, characterize, and monitor these viruses at the human–animal–environment interface. Nevertheless, major gaps in knowledge remain, particularly regarding the ecological drivers of spillover events, the role of intermediate hosts, and the integration of genomic data with epidemiological and ecological information within a One Health framework [1,2].

Whole genome sequencing (WGS) has become a cornerstone of modern coronavirus research and surveillance, enabling high-resolution analyses of viral evolution, transmission dynamics, and the emergence of variants of concern [3,4]. Despite its transformative impact, the full potential of WGS is often limited by fragmented surveillance systems and insufficient integration with clinical and epidemiological datasets. As a result, the translation of genomic insights into actionable public health and veterinary interventions remains uneven across regions and host species [5,6].

This Special Issue, “New Knowledge in the Study of Coronaviruses: Towards One Health and Whole Genome Sequencing Approaches, 2nd Edition”, was conceived to contribute to addressing these challenges by bringing together studies that apply genomic and multidisciplinary approaches to the investigation of coronaviruses in both human and animal contexts. The collected contributions highlight how sequencing-based strategies can enhance variant detection and characterization, improve data quality, and provide insights into viral evolution and selective pressures. In this context, studies published in this Special Issue illustrate the complementary value of WGS and molecular diagnostics for variant tracking, the challenges posed by protein-level evolutionary drift, and the importance of sequence quality assessment for reliable downstream analyses [7,8,9,10].

Taken together, the findings presented in this Special Issue reinforce the importance of embedding genomic surveillance within a broader One Health vision that integrates virology, veterinary science, ecology, and public health. Such an approach is essential not only for understanding ongoing coronavirus circulation, but also for strengthening preparedness and early warning systems for future emergence events. These perspectives align with broader international efforts emphasizing standardized sequencing, open data sharing, and interdisciplinary collaboration as key pillars of effective pathogen surveillance [5,6,7,8].

Looking ahead, future research should prioritize the expansion of WGS-based surveillance to under-sampled geographic regions and host species, particularly wildlife and domestic animals that may act as reservoirs or intermediate hosts. Harmonization of sequencing protocols, metadata standards, and analytical pipelines will be critical to ensure comparability and interoperability across studies. Moreover, closer integration between genomic surveillance and risk assessment frameworks will be necessary to translate molecular data into predictive models and evidence-based policy decisions. Sustained investment in One Health-oriented research and infrastructure will be crucial to improving global readiness for current and future coronavirus threats.

In conclusion, this Special Issue provides an updated and multifaceted overview of contemporary advances in coronavirus research, with particular emphasis on whole genome sequencing and One Health approaches. By bringing together complementary studies across disciplines and host systems, it highlights both the progress achieved and the challenges that remain. We hope that these contributions will inspire further research and collaboration aimed at strengthening integrated surveillance, preparedness, and response strategies for coronaviruses.
